# Influence of sex, age and thyroid function indices on dual-energy computed tomography-derived quantitative parameters of thyroid in patients with or without Hashimoto’s thyroiditis

**DOI:** 10.1186/s12880-023-00983-x

**Published:** 2023-02-05

**Authors:** Di Geng, Yan Zhou, Guo-Yi Su, Yan Si, Mei-Ping Shen, Xiao-Quan Xu, Fei-Yun Wu

**Affiliations:** 1 Department of Radiology, The First Affiliated Hospital of Nanjing Medical University, No. 300, Guangzhou Road, Nanjing, People’s Republic of China; 2 Department of Thyroid Surgery, First Affiliated Hospital of Nanjing Medical University, Nanjing, People’s Republic of China

**Keywords:** Thyroid, Hashimoto’s thyroiditis, Iodine concentration, Total iodine content, Dual-energy computed tomography

## Abstract

**Purpose:**

To study the influence of sex, age and thyroid function indices on dual-energy computed tomography (DECT)-derived quantitative parameters of thyroid in patients with or without Hashimoto’s thyroiditis (HT).

**Material and methods:**

A total of 198 consecutive patients who underwent DECT scan of neck due to unilateral thyroid lesions were retrospectively enrolled. Iodine concentration (IC), total iodine content (TIC) and volume of normal thyroid lobe were calculated. Influences of sex, age and thyroid function indices on DECT-derived parameters in overall study population, subgroup patients with, and those without HT were assessed using Mann–Whitney U test, Student’s T-test, and Spearman correlation analyses, respectively, as appropriate.

**Results:**

HT group showed significantly lower IC and TIC, while higher volume than No-HT group (all *p* < 0.001). The volume was larger in male than that in female in overall study population and No-HT group (*p* = 0.047 and 0.010, respectively). There was no significant difference in any DECT-derived parameters between low (≤ 35 years) and high (> 35 years) age group in all three groups (all *p* > 0.05). TPOAb and TgAb correlated positively with IC and TIC, and negatively with volume in overall study population (all *p* < 0.05). TPOAb and TgAb also correlated positively with IC in HT group (*p* = 0.002 and 0.007, respectively).

**Conclusion:**

DECT-derived parameters of thyroid differed significantly between patients with and without HT. Sex and thyroid function indices could affect the DECT-derived parameters. Aforementioned physiological factors should be considered when analyzing the DECT-derived parameters of thyroid.

## Introduction

Iodine is an essential microelement that was closely associated with the synthesis of thyroid hormone [1]. Several thyroid disorders can lead to the abnormal iodine intake and metabolism, and subsequently result in the changes of the iodine concentration (IC) and total iodine content (TIC) within the thyroid gland (TG) [2, 3]. Previously, Binh et al. have reported that the IC of TG correlated negatively with the iodine uptake at 3 h after oral administration of ^123^I in patients with Graves disease [4]. It indicates that accurately assessing the IC of TG can effectively assist in evaluating the iodine turnover, and then predicting the curative effect. In the past, iodine uptake and function was usually assessed using thyroid scintigraphy, however radioactive iodine is needed for this method [5]. To find a relatively simple and robust method for quantifying IC and TIC of the TG is urgently needed in clinical practice.

Dual-source dual-energy computed tomography (DECT), equipped with two X-ray tubes producing two different X-ray spectra, enables iodine-water material decomposition and provides a material-specific imaging method called iodine map [6, 7]. Previous phantom studies have reported that there were strong correlations between IC measured by DECT and true IC [8, 9]. Although Iida et al. have reported a linear relationship between CT number and IC of the TG [10], the effects of density and effective atomic number of tissue constituent on Hounsfield unit values should not be ignored [11]. By contrast, measuring IC based on DECT-derived iodine map might assess iodine nutritional status of thyroid more accurately.

Recently, the influences of physiological factors such as age, sex and thyroid functional status on CT or DECT-derived parameters have been studied increasingly [12–15]. Yi et al. has reported a positive correlation between thyroid CT density and age in children [12]. Meanwhile, Li et al. has found that the TIC in TG measured using DECT correlated positively with free triiodothyronine (FT3), but correlated negatively with thyroid-stimulating hormone (TSH), while the mean IC of TG was positively correlated with both FT3 and total triiodothyronine (TT3) in euthyroid patients [15]. Besides that, Rho et al. has reported that some diffuse thyroid disease (e.g. Hashimoto’s thyroiditis [HT]) could also result in inhomogeneous low attenuation of thyroid on CT images due to hypo-function [16]. However, to the best of our acknowledge, the study evaluating the influence of physiological factors (e.g. sex, age, thyroid function indices) on the volume, IC, and TIC in TG measured using DECT in both healthy subjects and patients with diffuse thyroid diseases is still lacked until now.

Therefore, this study aimed to evaluate the influence of sex, age and thyroid function indices on these DECT-derived quantitative parameters in patients with or without HT.

## Material and methods

### Study population

This study was approved by the local institutional review board of our hospital. The requirement of written informed consent was waived because of its retrospective nature. From January to December 2020, 837 consecutive patients who underwent DECT scan of neck due to suspected malignant nodular at our institution were retrospectively reviewed. We included the study population according to the following inclusion criteria: (1) patients with ultrasound-confirmed unilateral thyroid nodule, and with normal contralateral thyroid for further measurement; (2) patients without history of thyroid surgery or radioiodine therapy before ultrasound and DECT examination; (3) patients with available thyroid function results (e.g. FT3, free thyroxine [FT4], TSH, thyroid peroxidase antibody [TPOAb], and thyroglobulin antibody [TgAb]) tested within one week interval of DECT; (4) the imaging quality of DECT was adequate for subsequent analysis.

Finally, a total of 198 patients were enrolled in this study including 116 patients without HT (No-HT) and 82 patients with HT (Table 1). The diagnosis of HT was based on the pathological examination or the combination of clinical features, anti-thyroid antibodies and ultrasonographic characteristics [17]. Finally, there were 190 patients underwent puncture biopsy or surgery subsequently and obtained pathological specimens. Among 82 patients with HT, 20 patients were diagnosed based on the combination of clinical findings, levels of thyroid function parameters (mainly to TPOAb and TgAb), and patterns on ultrasound by an endocrinologist with 19 years of experience. As to the remaining 62 patients, the diagnosis of HT was established based on surgical pathology (60 patients obtained by subsequent postoperative pathology and 2 patients obtained by subsequent biopsy pathology).

**Table 1:** Clinical and ultrasound imaging features of patients in overall study population, HT and No-HT group

Variables	Overall study population (n = 198)	HT (n = 82)	No-HT (n = 116)	*p*
*Gender*				0.048
Male	38 (19.2%)	9 (11.0%)	29 (25.0%)	
Female	160 (80.8%)	73 (89.0%)	87 (75.0%)	
*Age (years)*				
Median (interquartile range)	34.0 (29.0–40.0)	34.0 (30.0–40.0)	34.5 (28.0–41.0)	0.854
≤ 35	109 (55.1%)	44 (53.7%)	65 (56.0%)	0.947
> 35	89 (44.9%)	38 (46.3%)	51 (44.0%)	
*Thyroid gland size (mm)*				
Vertical diameter	51.0 (47.0–55.0)	52.5 (48.0–56.0)	50.0 (46.0–54.0)	0.018
Transverse diameter	16.0 (14.0–18.0)	17.0 (15.0–18.0)	15.5 (13.8–17.0)	0.014
Anteroposterior diameter	14.0 (12.0–15.0)	14.0 (13.0–16.0)	13.0 (12.0–14.0)	< 0.001
*Thyroid nodule position*				0.251
Left lobe	96 (48.5%)	34 (41.5%)	62 (53.4%)	
Right lobe	102 (51.5%)	48 (58.5%)	54 (46.6%)	
*Thyroid nodule quantity*				0.993
Single	144 (72.7%)	60 (73.2%)	84 (72.4%)	
Multiple	54 (27.3%)	22 (26.8%)	32 (27.6%)	
*Thyroid nodule size (mm)*				
Vertical diameter	11.0 (7.3–17.0)	12.0 (7.4–17.8)	10.5 (7.1–15.0)	0.356
Transverse diameter	9.0 (6.3–13.0)	9.0 (6.3–14.0)	9.0 (6.3–12.0)	0.835
Anteroposterior diameter	8.6 (6.0–11.3)	8.7 (5.9–12.0)	8.4 (6.0–11.0)	0.863
TPOAb (IU/ml)	12.2 (8.4–23.2)	10.0 (8.0–13.1)	33.3 (10.6–127.0)	< 0.001
TgAb (IU/ml)	13.5 (10.0–131.4)	10.0 (10.0–13.2)	176.4 (42.0–368.7)	< 0.001
TPOAb positivity	39 (20.3%)	38 (48.1%)	1 (0.9%)	< 0.001
TgAb positivity	50 (25.5%)	50 (61.7%)	0 (0.0%)	< 0.001

Continuous and categorical variables are expressed as median (interquartile range) and absolute frequency (percentage), respectively. In overall study population, TPOAb was available in 192 patients and TgAb was available in 196 patients. In patients with HT, TPOAb was available in 79 patients and TgAb was available in 81 patients. In patients without HT, TPOAb was available in 113 patients and TgAb was available in 115 patients. HT, Hashimoto’s thyroiditis; No-HT, patients without HT

### DECT examination and post-processing

All examinations were performed on a third-generation dual-source CT scanner (SOMATOM Force, Siemens Healthineers, Germany), with the following parameters: gantry rotation time, 0.5 s; pitch, 0.7; field of view, 252 × 252 mm; tube A voltage and current, 80 kVp and 118 mAs; tube B voltage and current, Sn150 kVp and 59 mAs. DECT were scanned raging from skull base to thoracic inlet. Four-dimensional automatic real-time dose adjustment (CARE Dose 4D) and a third-generation advanced modeled iterative reconstruction (ADMIRE) technique were used for dose optimization.

### Imaging measurement

DECT data was transferred to a dedicated workstation (Syngo Via workstation, Siemens Healthineers, Germany), and post-processed by using a commercially available software (Syngo Dual Energy, version VB10B; Siemens Healthcare) with the three-material decomposition algorithm. The normal thyroid lobe without thyroid nodule was outlined manually in each slice. The volume of normal thyroid lobe was calculated by multiplying the sum of area in each section with the slice thickness. Meanwhile, three regions of interest (ROIs) (about 50 mm^2^) were delineated manually on three (the largest, neighbour upper, and neighbour under) layers of the normal thyroid lobe. The extent of ROIs was slightly smaller than the border of thyroid lobe for avoiding the influence of partial volume effect. After ROIs were placed, the IC would be automatically obtained. The TIC was then calculated using the following equation: TIC = IC × volume. All quantitative measurements were repeated three times, and the average values were used for further statistical analyses.

### Statistical analysis

Distribution for normality was evaluated using the Kolmogorov–Smirnov test. If normally distributed, quantitative parameters were reported as mean ± standard deviation (SD), and compared using student’s T-test. If not normally distributed, they would be presented as median (inter-quartile range, IQR), and compared using Mann–Whitney U test. Correlations between thyroid function and DECT parameters was analyzed by using Spearman correlation analyses. Due to non-normal distribution of age, thyroid gland size, thyroid nodule size and the value of TPOAb and TgAb value, they were expressed as median (inter-quartile range, IQR) and compared using Kruskal–Wallis test and subsequent post-hoc pairwise comparisons. Categorical variables were expressed as numbers and compared using Chi-square test or Fisher’s exact test. The intraobserver repeatability was evaluated using the first and second measurements conducted by reader 1. To ensure consistency in parameters measurement, another radiologist (with > 10 years of reading experience in head and neck radiology), as reader 2, also performed a measurement, including the IC, Volume and TIC of all patients. The interobserver repeatability was evaluated using the first measurement conducted by reader 1 and the measurement conducted conducted by reader 2. The intraclass correlation coefficient (ICC) was used to evaluate intraobserver and interobserver reproducibility. SPSS software (version 26.0, Chicago, USA) was used for statistical analyses. Two-tailed *p* < 0.05 was considered to indicate statistically significant difference.

## Results

### Clinical and ultrasound imaging features of patients

The clinical and ultrasound imaging features of overall study population, HT and No-HT groups are shown in Table 1. The largest nodule was selected when there were more than two nodules in unilateral thyroid lobe on ultrasound imaging. We observed differences in gender, thyroid gland size, autoantibodies’ level and positivity between three groups (all *p* < 0.050). However, there were no significant differences in age, thyroid nodule position, quantity and size between three groups (all *p* > 0.050).

### Result of consistency analysis

The intra- and interobserver repeatability of measuring DECT-derived parameters were excellent (all ICC > 0.75).

### Comparison of dual-energy CT parameters among patients with HT or No-HT

**Fig. 1:**
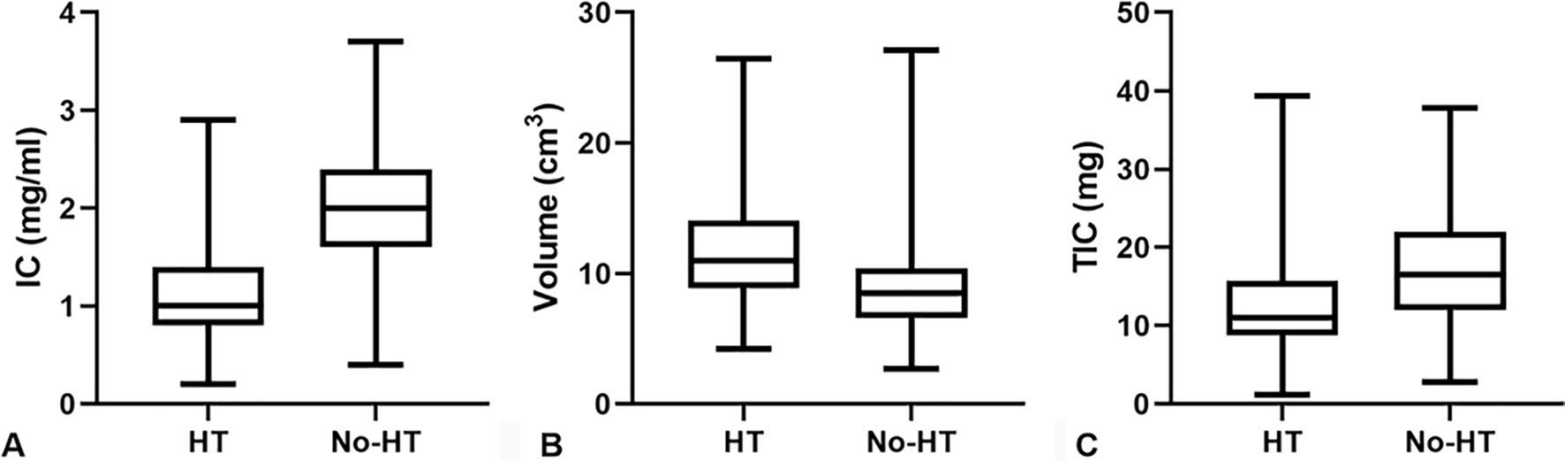
Box plots of IC (**A**), Volume (**B**) and TIC (**C**) among HT and No-HT patients. HT, Hashimoto’s thyroiditis; No-HT, patients without HT; IC, iodine concentration; TIC, total iodine content

**Fig. 2:**
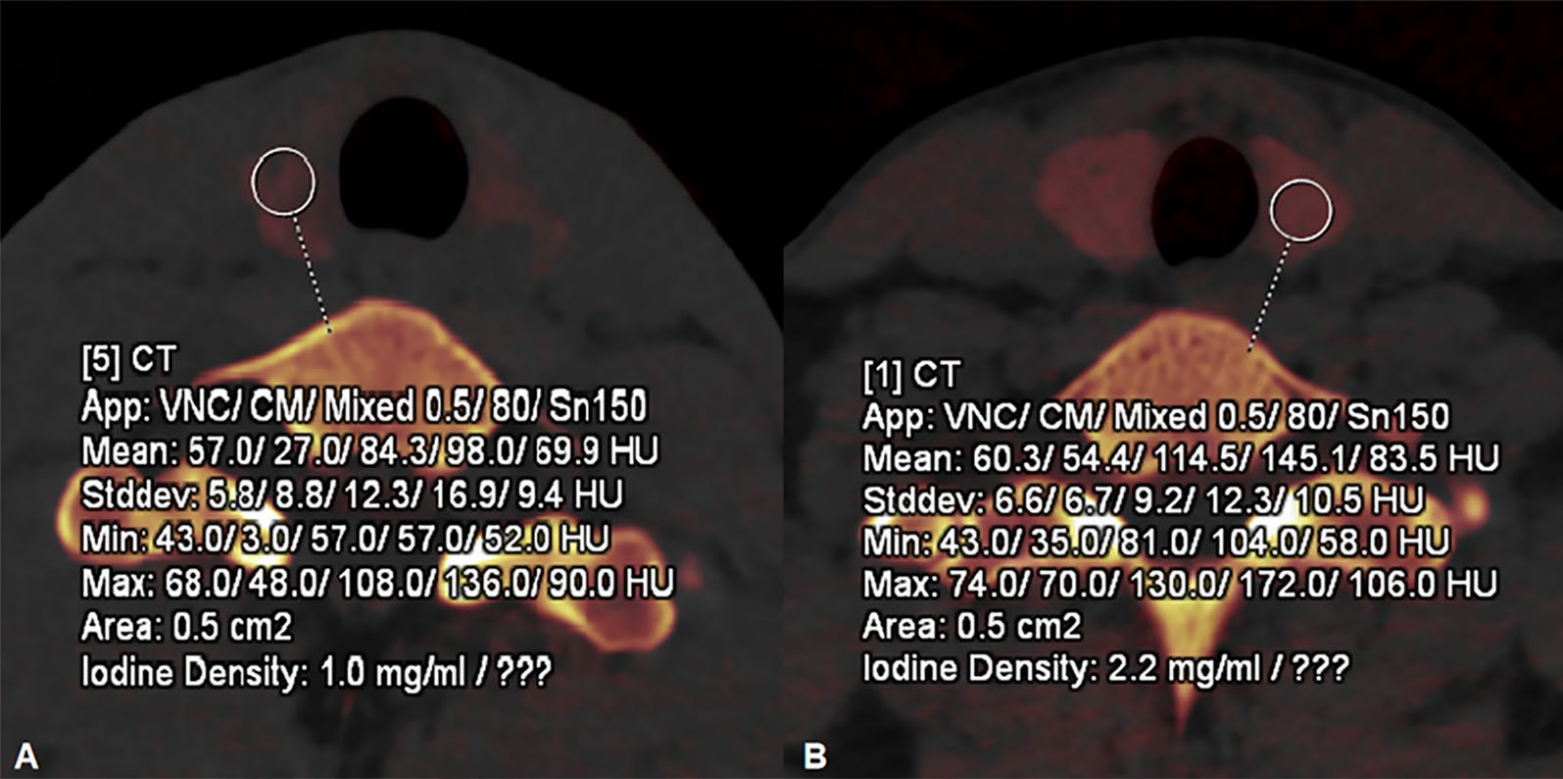
Representative figures of a 19-year-old male with HT (**A**) and a 28-year-old female without HT (**B**). After circular ROI was placed on the normal thyroid lobe, lower IC (1.0 mg/ml vs. 2.2 mg/ml) was found on HT

The comparisons of DECT parameters between HT and No-HT groups were summarized in Table 2. HT group showed significantly lower IC and TIC, and higher volume of normal thyroid lobe than No-HT group (all *p* < 0.001) (Fig. 1). Representative cases of one 19-year-old male with HT (A) and another 28-year-old female without HT were showed in Fig. 2. In addition, we compared the differences between thyroid vertical diameter, transverse diameter and anteroposterior diameter on ultrasound imaging of HT and No-HT groups. HT group had larger vertical diameter, transverse diameter and anteroposterior diameter in comparison to No-HT group (52.5 [48.0–56.0] vs. 50.0 [46.0–54.0], 17.0 [15.0–18.0] vs. 15.5 [13.8–17.0] and 14.0 [13.0–16.0] vs. 13.0 [12.0–14.0], *p* = 0.005, 0.004 and < 0.001, respectively).

**Table 2:** Comparison of DECT parameters between HT and No-HT patients

	HT (n = 82)	No-HT (n = 116)	*p*
IC (mg/ml)	1.00 (0.80–1.40)	2.00 (1.60–2.40)	< 0.001
Volume (cm^3^)	10.98 (8.85–14.03)	8.48 (6.58–10.39)	< 0.001
TIC (mg)	11.01 (8.76–15.70)	16.53 (11.96–21.96)	< 0.001

DECT, dual-energy computed tomography; HT, Hashimoto’s thyroiditis; No-HT, patients without HT; IC, iodine concentration; TIC, total iodine content

### Effect of sex on DECT parameters

**Fig. 3:**
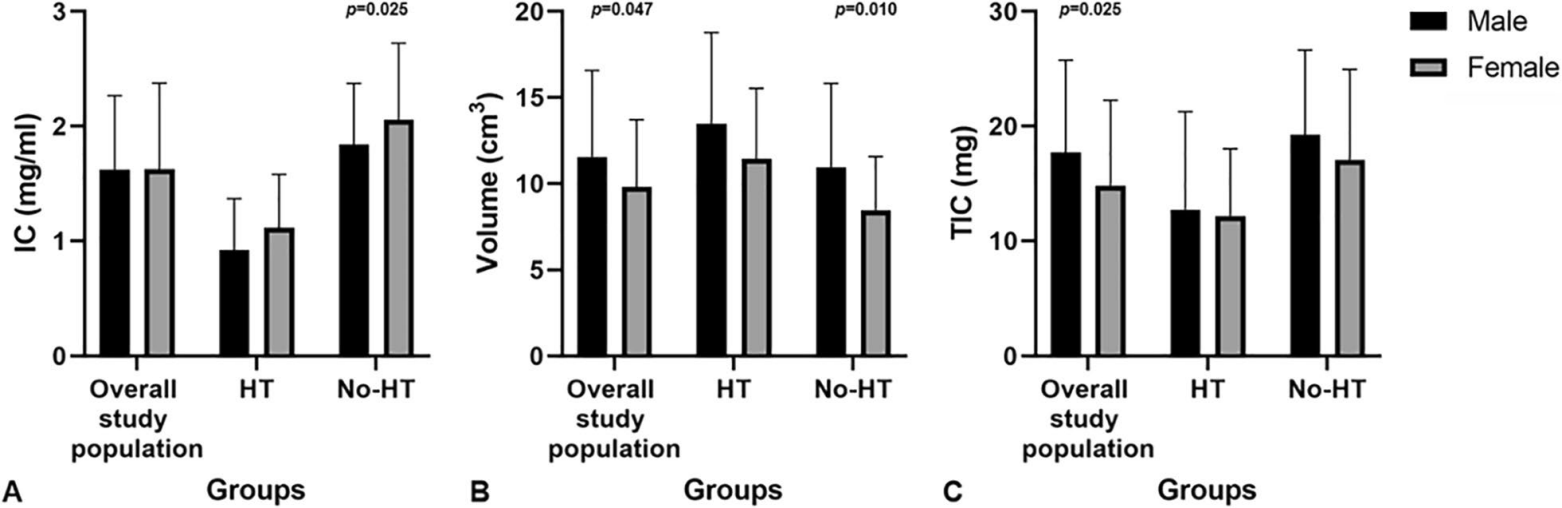
IC (**A**), Volume (**B**) and TIC (**C**) in overall study population, HT and No-HT group in male and female, plotted separately. The statistical comparisons were made between the male and female subjects within overall study population, HT and No-HT group. *p* values are not given when greater than 0.05. HT, Hashimoto’s thyroiditis; No-HT, patients without HT; IC, iodine concentration; TIC, total iodine content

DECT parameters of the thyroid lobe in the subgroup of male and female were showed and compared in Table 3. Male showed significantly higher volume of normal thyroid lobe than female in the overall study population (*p* = 0.047) and No-HT group (*p* = 0.010). Besides that, significant differences were also found in the TIC of overall study population (*p* = 0.025), and in the IC of No-HT group (*p* = 0.025), between male and female. However, no significant differences were found among any other sub-group comparisons (all *p* > 0.05) (Fig. 3).

**Table 3:** Effectof sex on DECT parameters

	Overall study population		*p1*	HT		*p2*	No-HT		*p3*
	Male (n = 38)	Female (n = 160)		Male (n = 9)	Female (n = 73)		Male (n = 29)	Female (n = 87)	
IC (mg/ml)	1.60 (1.20–1.90)	1.60 (0.93–2.20)	0.876	0.90 (0.55–1.20)	1.00 (0.80–1.40)	0.383	1.80 (1.50–2.15)	2.10 (1.70–2.50)	0.025
Volume (cm^3^)	10.70 (8.04–13.47)	9.23 (7.02–11.59)	0.047	12.35 (9.69–14.45)	10.92 (8.62–14.15)	0.282	9.26 (7.53–13.03)	8.31 (6.36–9.95)	0.010
TIC (mg)	16.15 (12.35–23.08)	13.60 (9.41–18.52)	0.025	12.51 (5.9–15.53)	10.89 (8.79–15.79)	> 0.999	19.26 ± 7.38	17.08 ± 7.87	0.192

DECT, dual-energy computed tomography; HT, Hashimoto’s thyroiditis; No-HT, patients without HT; IC, iodine concentration; TIC, total iodine content

### Effect of age on DECT parameters

**Fig. 4:**
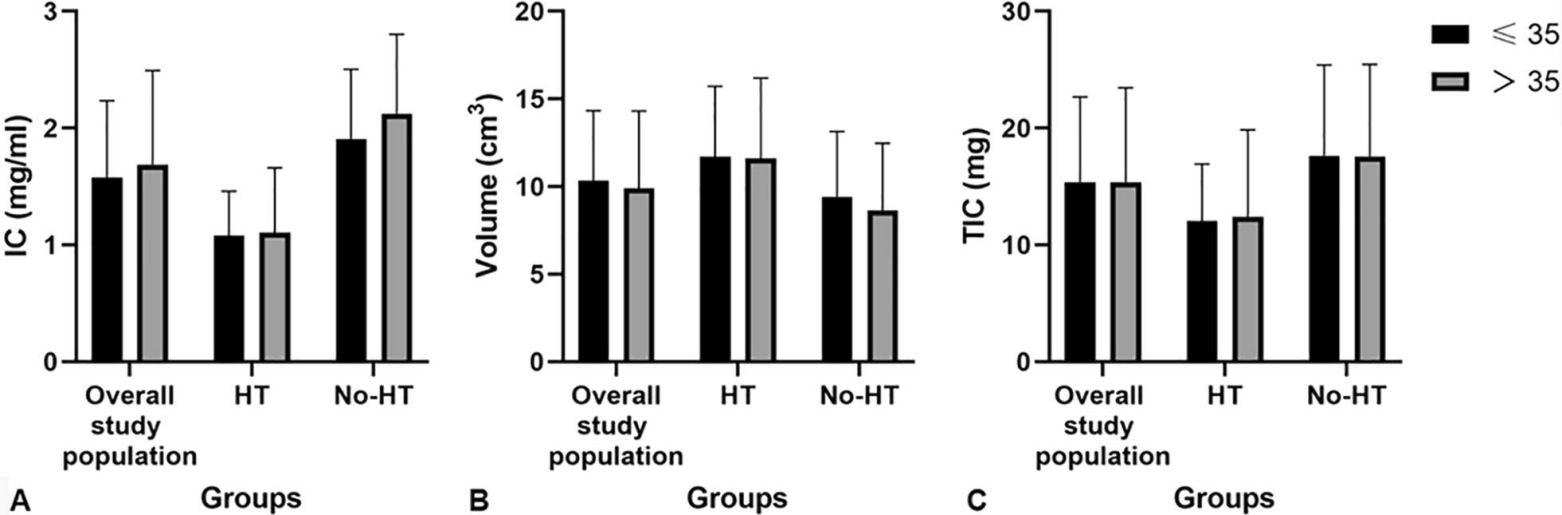
IC (**A**), Volume (**B**) and TIC (**C**) in overall study population, HT and No-HT group in low (≤ 35 years) and high (> 35 years) age subgroup, plotted separately. The statistical comparisons were made between the low and high age subjects within overall study population, HT and No-HT group. All *p* values are greater than 0.05. HT, Hashimoto’s thyroiditis; No-HT, patients without HT; IC, iodine concentration; TIC, total iodine content

DECT parameters of the thyroid lobe in the overall study population and in the subgroups stratified by patients age were exhibited and compared in Table 4. Among all three groups, there were no significant differences in IC, volume and TIC between different subgroups stratified by patients age (all *p* > 0.050) (Fig. 4).

**Table 4:** Effect of age on DECT parameters

	Overall study population		*p1*	HT		*p2*	No-HT		*p3*
	≤ 35 (n = 109)	> 35 (n = 89)		≤ 35 (n = 44)	> 35 (n = 38)		≤ 35 (n = 65)	> 35 (n = 51)	
IC (mg/ml)	1.50 (0.95–2.10)	1.60 (1.00–2.35)	0.417	0.95 (0.80–1.40)	1.05 (0.70–1.40)	0.786	1.91 ± 0.60	2.12 ± 0.68	0.073
Volume (cm^3^)	9.64 (7.91–12.22)	9.24 (6.70–11.78)	0.338	10.50 (8.91–15.28)	11.27 (8.14–13.65)	0.813	8.51 (6.88–10.71)	8.03 (6.30–10.22)	0.196
TIC (mg)	14.26 (10.00–18.90)	14.32 (9.83–19.70)	0.853	11.67 (8.78–15.57)	10.77 (8.54–16.92)	0.845	17.64 ± 7.76	17.60 ± 7.88	0.975

DECT, dual-energy computed tomography; HT, Hashimoto’s thyroiditis; No-HT, patients without HT; IC, iodine concentration; TIC, total iodine content

### Effect of thyroid function index on DECT parameters

**Fig. 5:**
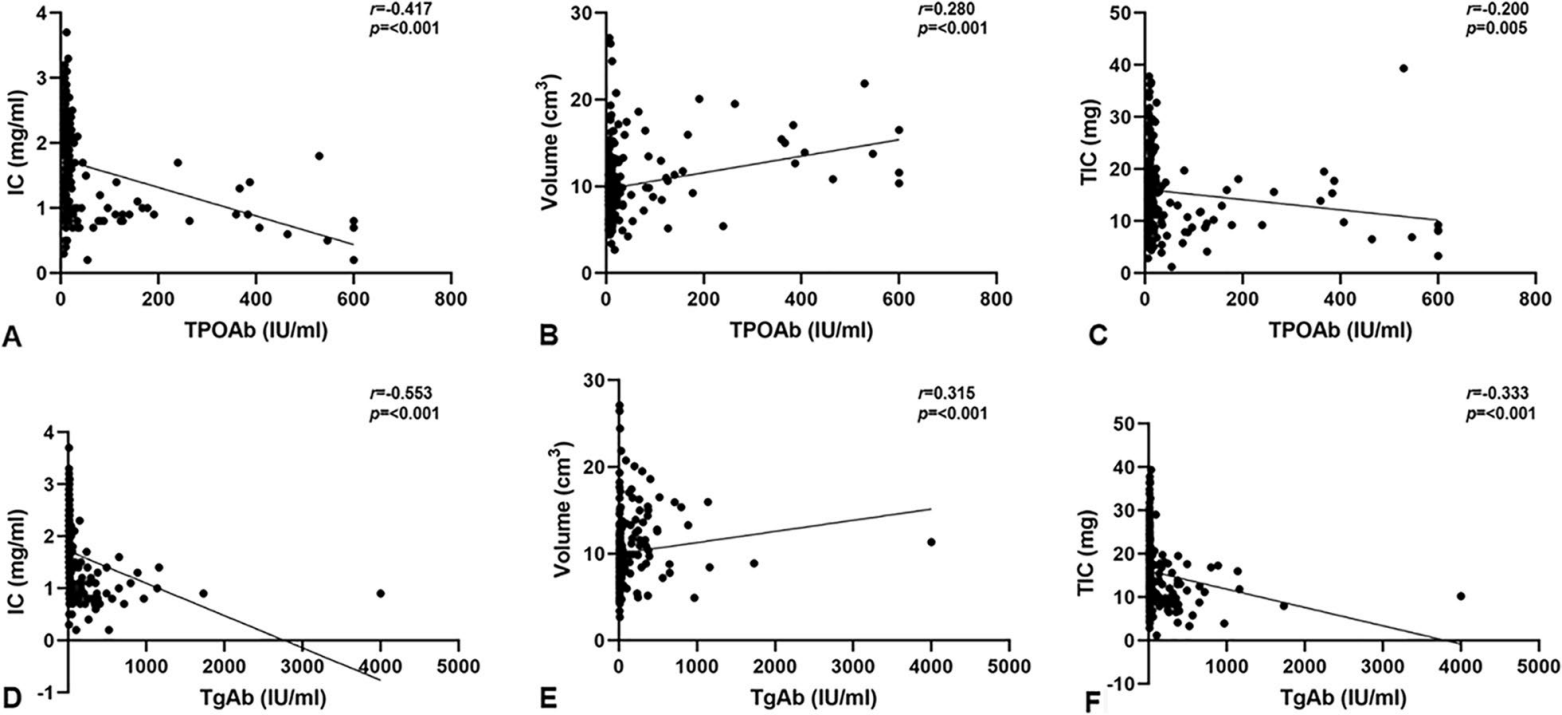
Correlation between TPOAb concentration and IC (**A**); TPOAb concentration and Volume (**B**); TPOAb concentration and TIC (**C**); TgAb concentration and IC (**D**); TgAb concentration and Volume (**E**); and TgAb concentration and TIC (**F**) in overall study population. IC, iodine concentration; TIC, total iodine content

In overall study population, FT3, FT4 and TSH were available in 197 patients, TPOAb was available in 192 patients, and TgAb was available in 196 patients. Both serum TPOAb and TgAb were negatively correlated with the IC (r = -0.417, -0.553, respectively, both *p* < 0.001) and TIC (r = − 0.200, *p* = 0.005; r = -0.333, *p* < 0.001, respectively), and positively correlated with the volume of thyroid lobe (r = 0.280, 0.315, respectively, both *p* < 0.001). However, no significant correlations were observed between IC, TIC or volume of thyroid lobe and the serum FT3, FT4, TSH (all *p* > 0.050) (Table 5, Fig. 5).

**Table 5:** Correlations between thyroid function indices and DECT parameters

	Overall study population			HT			No-HT		
	IC	Volume	TIC	IC	Volume	TIC	IC	Volume	TIC
*FT3*									
*p*	0.923	0.051	0.211	0.427	0.021	0.567	0.049	0.123	0.788
*r*	− 0.007	0.139	0.089	− 0.089	0.256	0.065	− 0.183	0.144	0.025
*FT4*									
*p*	0.863	0.059	0.093	0.811	0.158	0.381	0.062	0.012	0.326
*r*	0.012	0.135	0.120	− 0.027	0.158	0.099	− 0.174	0.233	0.092
*TSH*									
*p*	0.346	0.427	0.090	0.136	0.371	0.028	0.220	0.215	0.926
*r*	− 0.068	− 0.057	− 0.121	− 0.167	− 0.101	− 0.244	0.115	− 0.116	− 0.009
*TPOAb*									
*p*	< 0.001	< 0.001	0.005	0.002	0.053	0.183	0.345	0.049	0.360
*r*	− 0.417	0.280	− 0.200	− 0.336	0.218	− 0.151	− 0.090	0.186	0.087
*TgAb*									
*p*	< 0.001	< 0.001	< 0.001	0.007	0.428	0.164	0.577	0.626	0.971
*r*	− 0.553	0.315	− 0.333	− 0.297	0.089	− 0.156	0.052	0.046	0.003

DECT, dual-energy computed tomography; HT, Hashimoto’s thyroiditis; No-HT, patients without HT; IC, iodine concentration; TIC, total iodine content

In patients with HT, FT3, FT4, TSH and TgAb were available in 81 patients, and TPOAb was available in 79 patients. The IC was negatively correlated with TPOAb (r = -0.336, *p* = 0.002) and TgAb (r = -0.297, *p* = 0.007), while no correlations were found between IC and serum FT3, FT4 and TSH (all *p* > 0.050). Besides that, a positive correlation was also found between the volume of thyroid lobe and serum FT3 (r = 0.256, *p* = 0.021), and a negative correlation was found between the TIC and serum TSH (r = -0.244, *p* = 0.028). No significant correlations were found between any other sub-group correlation analysis (all *p* > 0.050) (Table 5).

In patients without HT, FT3, FT4, TSH were available in 116 patients, TPOAb was available on 113 patients, and TgAb was available on 115 patients. An inverse relationship was noted between IC and serum FT3 (r = -0.183, *p* = 0.049), and no association was observed between IC and any other thyroid function indices (all *p* > 0.050). There were positive correlations between volume of thyroid lobe and FT4 (r = 0.233, *p* = 0.012) as well as TPOAb concentrations (r = 0.186, *p* = 0.049), while no correlation was detected among serum FT3, TSH and TgAb (all *p* > 0.050). As to TIC, no significant correlation was found with all the thyroid function indices (all *p* > 0.050) (Table 5).

## Discussion

This retrospective study had several main findings. First, HT group showed significantly lower IC, TIC, and higher volume of normal thyroid lobe than No-HT group. Second, sex could affect the thyroid volume in the overall study population and No-HT group. Female usually showed lower volume of normal thyroid lobe than male. Third, age had no potential effect on all included DECT-derived parameters. Fourth, patients with increased TPOAb and TgAb concentrations tended to present lower IC and TIC, and higher volume of normal thyroid lobe.

HT patients showed lower IC and TIC values, but higher volume of normal thyroid lobe in present study. HT was characterized with chronic lymphocytic inflammation caused by a direct T-cell attack on the TG. During the disease process, anti-thyroglobulin and anti-thyroperoxidase antibodies against exposed thyroid antigens were continuously produced [18]. The high titers of thyroid autoantibodies caused direct toxic effects on thyrocytes to death, followed by lymphocytic infiltration and hyperplasia of thyroid follicles. In the end, the decreased iodine uptake due to gradual thyroidal damage, and the increased thyroid volume due to diffuse goiter inevitably occurred in most patients [19, 20]. These immunopathologic mechanisms might be the potential reasons for the differences of DECT parameters between HT and No-HT patients.

In this study, males were found to have higher volume of normal thyroid lobes than females in the overall study population and No-HT group, which was in agreement with previous studies measured by ultrasound [21, 22]. In other words, sex had an influence on the thyroid volume except in HT group. We speculated that, in the subgroup of HT, the thyroid volume was more closely associated with the disease process of HT itself. Besides that, female held a higher incidence of HT which was mostly characterized by goiter than male [23]. Summing up the above, the influence of sex was weakened in HT group.

The differences in DECT parameters between two age subgroups were not observed in our study, which indicated that age did not play an important role on IC, TIC and volume of normal thyroid lobe. Previously, Lee et al. reported a negative relationship between thyroid volume and age [24], while other surveys did not show significant correlation between decreasing thyroid volume and increasing age [21, 25]. In the future, larger scale study was needed to clarify, the relationship between thyroid volume and age. Additionally, Pandey et al. did not observe a significant correlation between thyroid CT density and age [14], which was similar to our results.

We found significant correlations between DECT parameters and TPOAb or TgAb concentrations, especially in the overall study population. Both two thyroid autoantibodies were produced by lymphocytes within the TG [26]. In previous studies, the titers of serum TPOAb and TgAb had been reported to be positively correlated with an increased inflammatory reaction. Both of them reflected the degree of lymphocytic infiltration in the gland, and had been proven to be a risk factor of hypothyroidism [27–29]. Thereby, the higher titers of serum TPOAb and TgAb would contribute to lower IC and TIC, while higher volume of normal thyroid lobe. In addition, our present study indicated that there was a positive correlation between the volume of normal thyroid lobe and serum FT3 level in HT group, which was consistent with the study carried out by Kawasaki et al. [30]. They reported that HT patients with large goiters tended to had relatively high serum FT3 levels, and they attributed this result to the elevation of thyroidal deiodinase activity in HT patients.

Previsous studies have indicated that DECT was useful in the preoperative differentiation between benign and malignant cervical lymph modes (LNs) in the patients with papillary thyroid cancer (PTC) [31, 32]. However, as we know, the proliferation of autoreactive T and B cells would lead to the reactive hyperplasia of cervical LNs in the patients with HT [33], which subsequently result in the enlarged size and increased enhancement in LNs showed on DECT. This situation would increase the difficulty in differentiating reactive from metastatic LNs, which might be associated with the higher number of dissected LNs while similar rate of malignant LNs in the patients with HT [34]. All these results showed that, being aware of the medical history of thyroiditis or thyroid function indicators was important when diagnosing the metastatic cervical LNs, however, these information were not always available when we are analyzing the DECT data. Our study found that DECT-derived quantitative parameters were affected by Hashimoto’s thyroiditis status and correlated with levels of anti-thyroid antibodies. DECT-derived parameters of thyroid could serve as a crucial reference for the radiologists to predict the thyroiditis status, and then individually and accurately evaluate the potential metastasis of LNs, thereby assisting the clinicians in establishing the surgical plans. Furthermore, this study also found that DECT-derived parameters were related to sex, but not to age, so the sex difference should be taken into account in the DECT imaging-based diagnosis.

There were several limitations in our study. First, this was a retrospective study with limited sample size. Further study with more study population was needed to confirm our results. Second, because of the ionizing radiation, we could only retrospectively enroll the consecutive patients who underwent DECT scan of neck due to unilateral thyroid lesions, and analyze the contralateral normal thyroid lobe. Although the thyroid lesions (mostly papillary thyroid carcinoma) usually did not markedly affect the thyroid function indices, potential influence might still exist.

## Conclusions

In conclusion, our study indicated that DECT-derived parameters of thyroid differed significantly between patients with and without HT. Sex and thyroid function indices could affect the DECT-derived parameters of thyroid. All the aforementioned physiological factors should be taken into account when analyzing the DECT- derived parameters of thyroid in clinical practice.

## Data Availability

The data that support the findings of this study are available from the corresponding author on reasonable request.
